# A genome-wide association study of asthma symptoms in Latin American children

**DOI:** 10.1186/s12863-015-0296-7

**Published:** 2015-12-03

**Authors:** Gustavo N. O. Costa, Frank Dudbridge, Rosemeire L. Fiaccone, Thiago M. da Silva, Jackson S. Conceição, Agostino Strina, Camila A. Figueiredo, Wagner C. S. Magalhães, Maira R. Rodrigues, Mateus H. Gouveia, Fernanda S. G. Kehdy, Andrea R. V. R. Horimoto, Bernardo Horta, Esteban G. Burchard, Maria Pino-Yanes, Blanca Del Rio Navarro, Isabelle Romieu, Dana B. Hancock, Stephanie London, Maria Fernanda Lima-Costa, Alexandre C. Pereira, Eduardo Tarazona, Laura C Rodrigues, Mauricio L. Barreto

**Affiliations:** Instituto de Saúde Coletiva, Universidade Federal da Bahia, Salvador, Brazil; Instituto de Matemática, Universidade Federal da Bahia, Salvador, Brazil; Instituto de Ciências da Saúde, Universidade Federal da Bahia, Salvador, Brazil; Instituto de Ciências Biológicas, Universidade Federal de Minas Gerais, Belo Horizonte, Brazil; Instituto do Coração, Universidade de São Paulo, São Paulo, Brazil; Programa de Pós Graduação em Epidemiologia, Universidade Federal de Pelotas, Pelotas, Brazil; Department of Medicine, University of California, San Francisco, USA; Department of Health and Human Services, Epidemiology Branch, National Institute of Environmental Health Sciences, National Institutes of Health, Research Triangle Park, North Carolina, USA; Instituto Nacional de Salud Publica, Cuernavaca, Mexico; Behavioral and Urban Health Program, Research Triangle Institute (RTI) International, Research Triangle Park, North Carolina, USA; Instituto de Pesquisas Rene Rachou, Fundação Oswaldo Cruz, Belo Horizonte, Brazil; Department of Non-communicable Disease Epidemiology, London School of Hygiene and Tropical Medicine, London, UK; Department of Infectious Disease Epidemiology, London School of Hygiene and Tropical Medicine, London, UK; Centro de Pesquisa Gonçalo Muniz, Fundação Osvaldo Cruz, Salvador, Brazil

**Keywords:** Asthma symptoms, Genome-wide association, Latin America, Children

## Abstract

**Background:**

Asthma is a chronic disease of the airways and, despite the advances in the knowledge of associated genetic regions *in recent years*, their mechanisms have yet to be explored. Several genome-wide association studies have been carried out in recent years, but none of these have involved Latin American populations with a high level of miscegenation, as is seen in the Brazilian population.

**Methods:**

1246 children were recruited from a longitudinal cohort study in Salvador, Brazil. Asthma symptoms were identified in accordance with an International Study of Asthma and Allergies in Childhood (ISAAC) questionnaire. Following quality control, 1 877 526 autosomal SNPs were tested for association with childhood asthma symptoms by logistic regression using an additive genetic model. We complemented the analysis with an estimate of the phenotypic variance explained by common genetic variants. Replications were investigated in independent Mexican and US Latino samples.

**Results:**

Two chromosomal regions reached genome-wide significance level for childhood asthma symptoms: the 14q11 region flanking the *DAD1* and *OXA1L* genes (rs1999071, MAF 0.32, OR 1.78, 95 % CI 1.45–2.18, *p*-value 2.83 × 10^−8^) and 15q22 region flanking the *FOXB1* gene (rs10519031, MAF 0.04, OR 3.0, 95 % CI 2.02–4.49, *p*-value 6.68 × 10^−8^ and rs8029377, MAF 0.03, OR 2.49, 95 % CI 1.76–3.53, *p*-value 2.45 × 10^−7^). eQTL analysis suggests that rs1999071 regulates the expression of *OXA1L* gene. However, the original findings were not replicated in the Mexican or US Latino samples.

**Conclusions:**

We conclude that the 14q11 and 15q22 regions may be associated with asthma symptoms in childhood.

**Electronic supplementary material:**

The online version of this article (doi:10.1186/s12863-015-0296-7) contains supplementary material, which is available to authorized users.

## Background

Asthma is classified as a complex and inflammatory disease of the respiratory tract with distinct phenotypes and has a major impact on mortality, morbidity and quality of life. However, the geographical area in which it occurs should be taken into account in order to reflect on its complexity. It has been occurring increasingly in Latin America and a number of authors attribute a part of this rise to the social and urban inequalities present in these countries [1].

Recent reviews suggest that a significant amount of childhood asthma could be attributed to genetic inheritance [2]. A considerable number of studies on candidate genes have been carried out in recent years, based on an immunological understanding of asthma, in an attempt to understand the genetic mechanisms of asthma, but inconsistent replication suggested that these studies mostly reported false-positive results [3]. A further important observation is that the studies on association between genetics and asthma were predominantly developed in populations of North American and European origin^4^, where the profile of disease differs from the asthma established in Latin American populations.

The use of Genome-Wide Association Studies (GWAS) as an alternative to candidate gene association analyses has become possible with the development of genomic analysis techniques. GWAS is a form of studying genetic association in which hundreds of thousands of single nucleotide polymorphisms (SNPs) are evaluated through relations with a specific phenotype, without a previous causal hypothesis [4].

The first GWAS of asthma identified various markers in the 17q21 region, with common variants that appear to contribute to a substantial proportion of asthma cases in the group of children investigated [5]. Later studies revealed that this region is important not only for asthma in children and highlighted the importance of other genes such as the chromosome 18 cluster *IL1RL1/IL18R1* in adults [6] and *PDE11A* in children [7], among others. In turn, GWAS in non-white populations have indicated different SNPs for asthma, such as *ADRA1B*, *RPPN*, and *DPP10* [8].

This study differs from others as it considers an extremely admixed population, which does not correspond to the USA-Europe axis and seeks to understand the genetic basis of asthma symptoms using genome-wide techniques. The potential advantages of this approach are higher frequencies of some disease SNPs, greater extent of linkage disequilibrium due to admixture and increased effect sizes for SNPs in the presence of certain environmental risk factors, for example, changes in diet, physical activity, exposure to allergens, indoor pollutants and psychosocial factors [1].

This study aims to explore the effects of genetic markers on asthma symptoms in a population of children living in the city of Salvador, Brazil by means of a GWAS. We then assessed the heritability in this population and investigated the possible metabolic pathways associated with asthma symptoms.

## Results

After quality control, 1246 children aged 5 to 12 years old were analysed. 673 of these were male and 573 female. From this total, 280 (22 %) presented asthma symptoms which were defined as cases, 55.5 % male and 44.5 % female. The others 966 (78 %) without asthma symptoms was defined as controls, 53.6 % male and 46.3 % female.

### Association test

**Fig. 1:**
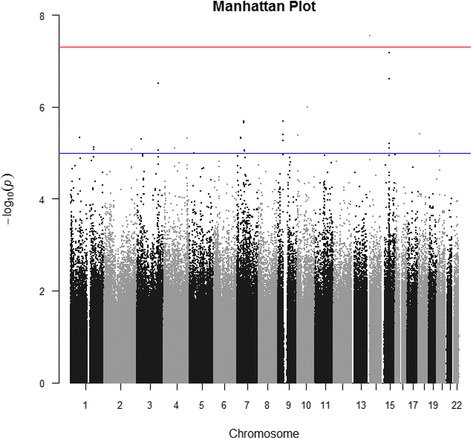
Manhattan plot for asthma symptoms in children, adjusted for population structure

**Fig. 2:**
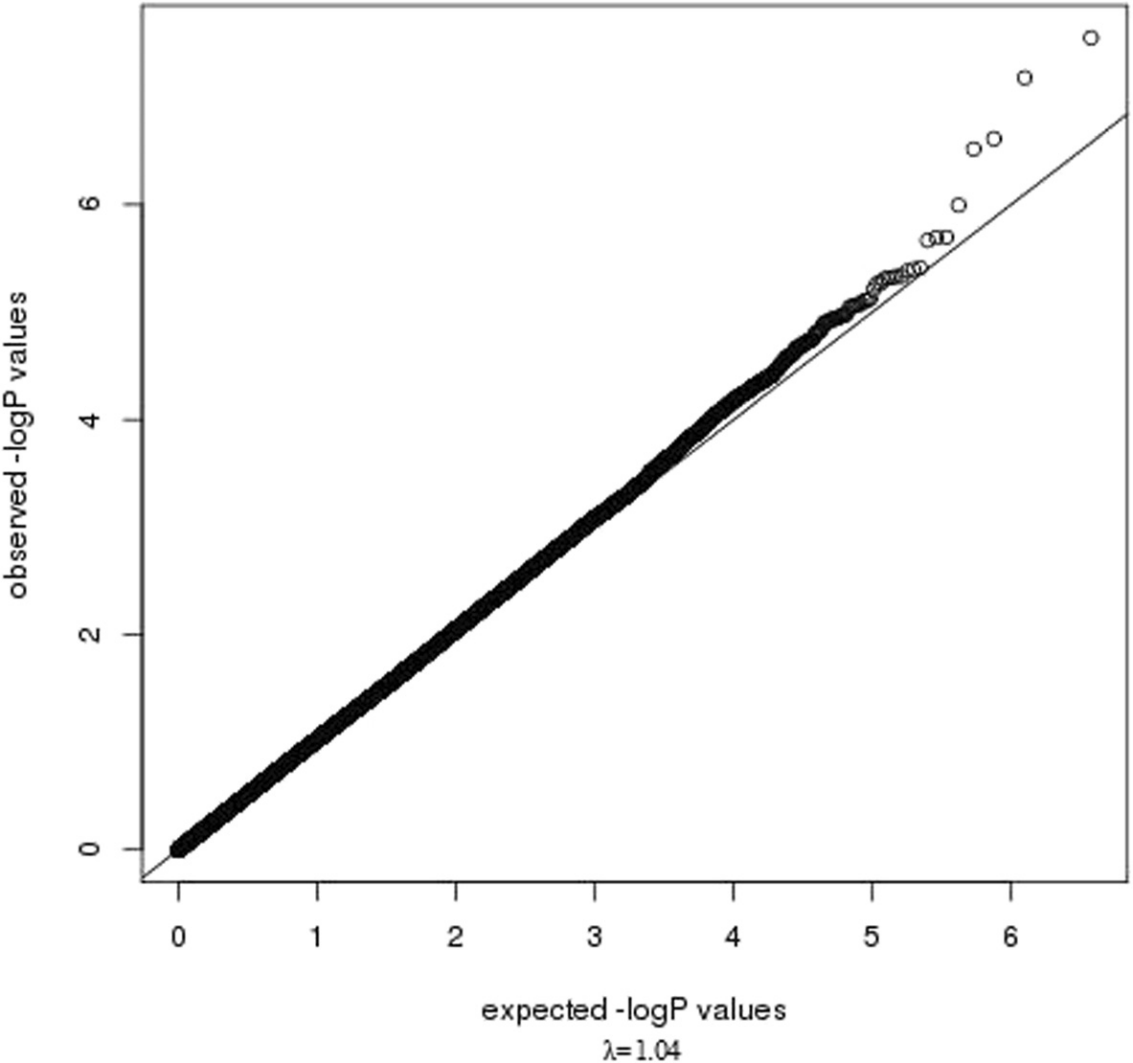
QQ-plot for childhood asthma symptoms, adjusted for population structure

**Fig. 3:**
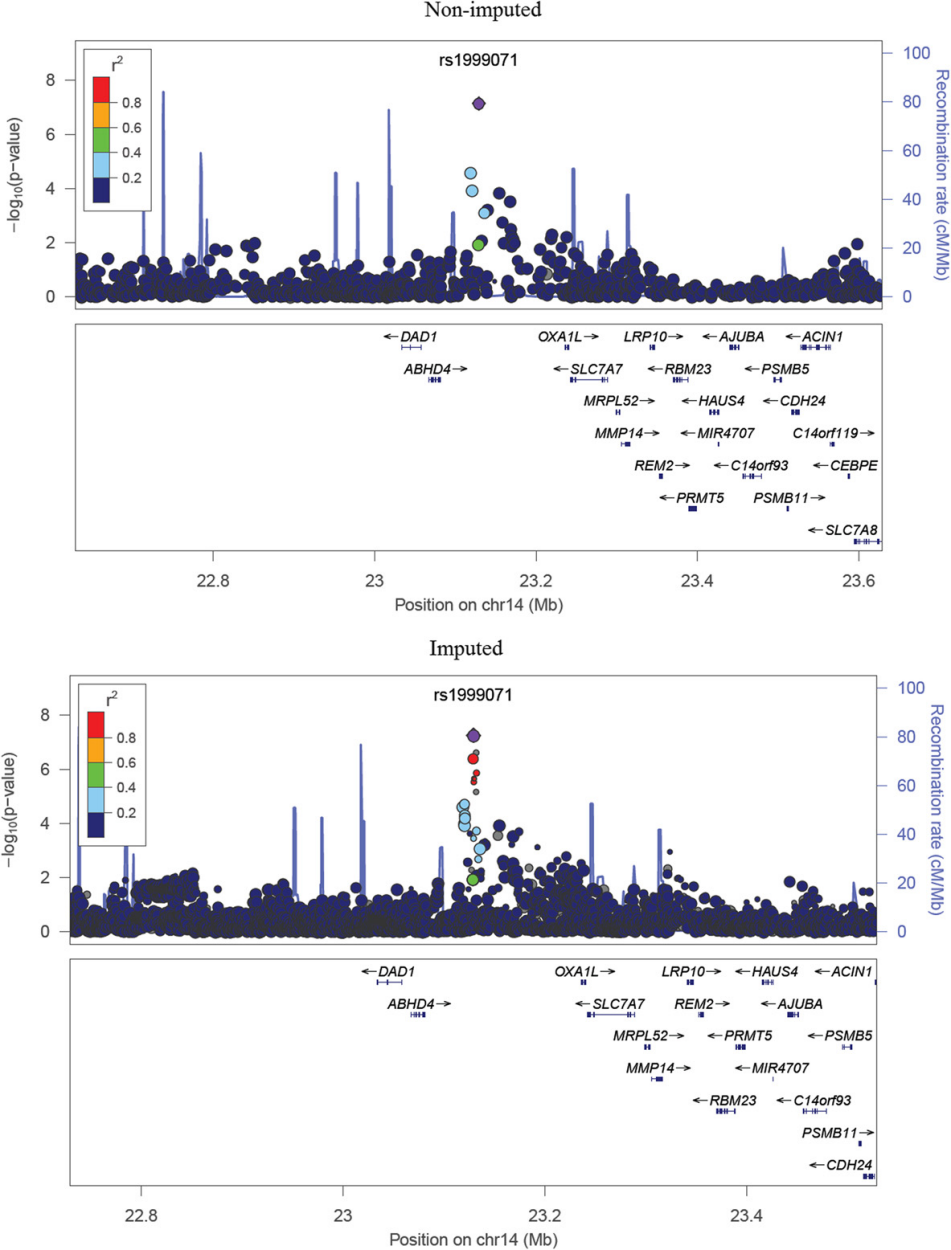
Regional plot of chromosome 14, which is the region most associated with childhood asthma symptoms

**Fig. 4:**
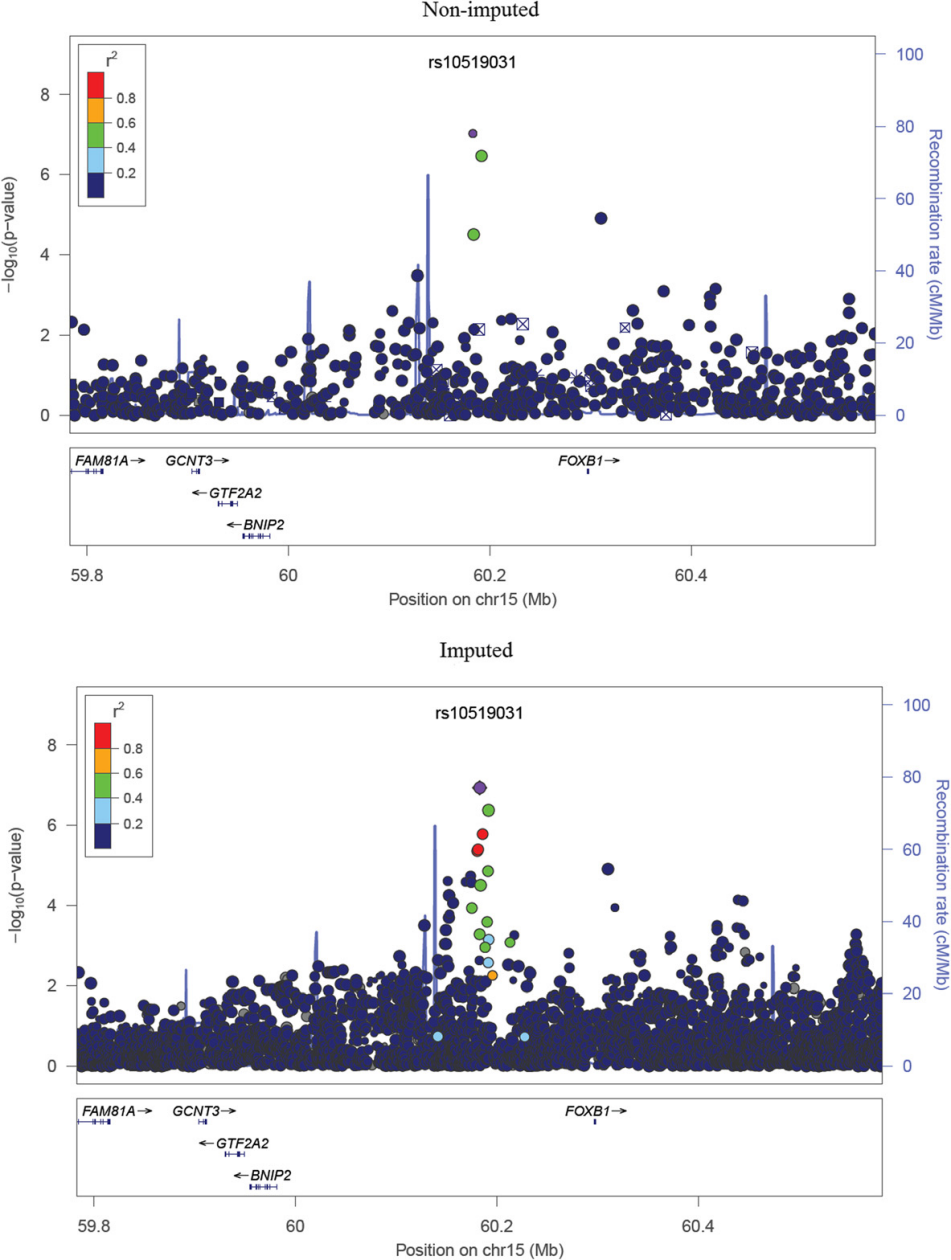
Regional plot of chromosome 15, which is the second region most associated with childhood asthma symptoms

Following a PCA adjustment for ancestry (Additional file 1: Figure S1), the genomic inflation factor (λ) was 1.04, indicating a low probability of false-positive associations as a result of population structure. The most strongly associated SNPs were found on chromosome 14 (region 14q11, Fig. 1), rs1999071 variant (OR: 1.78; 95 % CI: 1.45–2.18; *p*-value: 2.83 × 10^−8^) in the intergenic region of 100 kb up-stream to the *OXA1L* (*oxidase (cytochrome c) assembly 1-like*) gene. The second most associated chromosome region was 15q21, specifically SNPs rs10519031 (OR: 3.0; 95 % CI: 2.02–4.49; *p*-value: 6.68 × 10^−8^) and rs8029377 (OR: 2.49; 95 % CI: 1.76–3.53; *p*-value: 2.45 × 10^−7^), both in an intergenic region. Table 1 lists the 20 most significant SNPs (for further information, see Additional file 1: Table S2). The quantile-quantile plot revealed some deviations in the tail, but not systematic deviation, indicating that SNPs which are genuinely associated with asthma symptoms could be present (Fig. 2). Following imputation for chromosomes 14 and 15, we observed that the associated SNPs with greater statistical significance remained and were identified as belonging to the regions flanking the *DAD1* and *OXA1L* genes in chromosome 14q11 and *FOXB1* in chromosome 15q21 (Figs. 3 and  4).

**Table 1:** The 20 SNPs which are most associated with asthma, corrected by the first three principal components for ancestry

Rank	Chromosome	SNP	Position (bp)	Risk Allele	MAF	Gene	*Odds ratio*	IC (95 %)	*p*
1	14	rs1999071	23129207	C	0.32	Intergenic	1.78	(1.45 – 2.18)	2.834 × 10^−08^
2	15	rs10519031	60183005	C	0.04	Intergenic	3.01	(2.02 – 4.49)	6.676 × 10^−08^
3	15	rs8029377	60191985	C	0.06	Intergenic	2.49	(1.76 – 3.53)	2.454 × 10^−07^
4	3	rs77165709	159452876	T	0.08	IQCJ-SCHIP1	2.27	(1.66 – 3.18)	3.045 × 10^−07^
5	10	rs10159952	77562470	A	0.09	C10orf11	2.04	(1.53 – 2.72)	1.016 × 10^−06^
6	9	rs1329568	37037976	T	0.11	LOC100130458	1.93	(1.47 – 2.54)	2.02 × 10^−06^
7	7	rs1425883	49754984	T	0.36	Intergenic	0.60	(0.49 – 0.74)	2.037 × 10^−06^
8	7	rs1543902	49754752	G	0.36	Intergenic	0.60	(0.49 – 0.74)	2.164 × 10^−06^
9	18	rs76227669	10787366	T	0.02	PIEZO2	4.06	(2.24 – 7.36)	3.881 × 10^−06^
10	9	rs4878674	37036329	T	0.12	Intergenic	1.90	(1.45 – 2.49)	4.027 × 10^−06^
11	10	rs1244495	7881339	G	0.38	TAF3	0.61	(0.50 – 0.75)	4.169 × 10^−06^
12	1	rs269330	68548590	T	0.16	GNG12-AS1	1.75	(1.38 – 2.22)	4.669 × 10^−06^
13	7	rs10268364	28708465	C	0.36	CREB5	1.59	(1.30 – 1.94)	4.671 × 10^−06^
14	7	rs41335	28704468	C	0.49	CREB6	0.64	(0.52 – 0.77)	4.793 × 10^−06^
15	4	rs72998173	173715118	G	0.14	GALNTL6	1.76	(1.38 – 2.24)	4.848 × 10^−06^
16	3	rs4373023	34077223	T	0.43	Intergenic	1.57	(1.29 – 1.91)	4.977 × 10^−06^
17	9	rs1329567	37038326	A	0.09	LOC100130458	1.97	(1.47 – 2.64)	5.46 × 10^−06^
18	9	rs2381598	37041246	C	0.09	Intergenic	1.97	(1.47 – 2.64)	5.474 × 10^−06^
19	15	rs12901887	56941976	G	0.23	ZNF280D	0.56	(0.43 – 0.72)	6.224 × 10^−06^
20	1	rs79530846	171950308	T	0.03	vDNM3	3.08	(1.88 – 5.03)	7.542 × 10^−06^

**Fig. 5:**
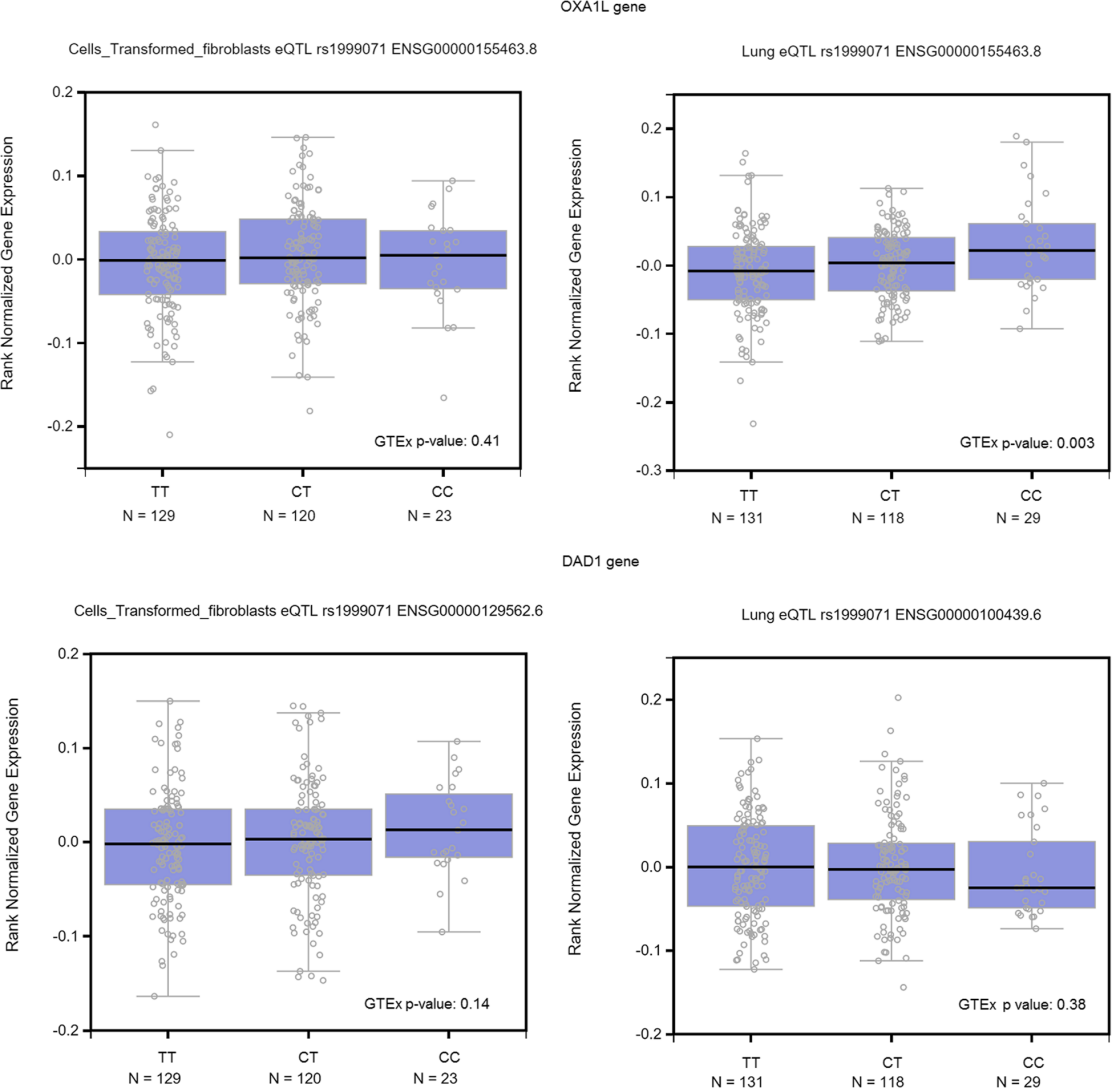
Expression of genes *OXA1L* and *DAD1*, that flanking the rs1999071, in chromosome 14

We examined whether rs1999071 is associated with differential expression of DAD1 and OXA1L in chromosome 14 using the GTEx browser [9] in lung tissue and transformed fibroblast cells (Fig. 5). We found differential expression of *OXA1L* in lung tissue (GTEX *p*-value: 0.003).

For replication in GALA II and MCCAS, we provided a list of 75 SNPs, in which 25 were the most associated in the initial analysis, to which were added the most associated SNPs in the 14q11 and 15q21 regions after imputation (25 SNPs for each chromosome). In GALA II, 65 SNPs were available (Additional file 1: Table S3), but only one SNP in chromosome 10, rs10159952, was replicated (OR: 1.37; 95 % CI: 1.07–1.76; *p*-value: 0.01). This SNP is an intronic variant in the *C10orf11* gene and remained associated after combined analysis (OR _combined_: 1.63; 95 % CI: 1.35–1.97; *p*-value _combined_: 4.03 × 10^−07^). In MCCAS, the data were available on 14 SNPs overall, however no SNP had a *P* value < 0.05 (Additional file 1: Table S3), and the combined *p*-value of rs10159962 was 3.25 × 10^−06^.

### The proportion of phenotypic variance explained by the genome

It is observed in Table 2 that 70 % of the total phenotypic variation (liability for asthma symptoms) was explained by the genotyped SNPs (*p*-value: 0.001). This variance dropped to 69 % with the removal of the 20 most associated SNPs and to 12 % in an analysis of the 20 most associated SNPs; however the standard errors on each of these values are large. In the analysis separated by chromosome, chromosomes 4, 7, 10, 13 and 15 were those which most explained asthma symptoms (Additional file 1: Figure S2).

**Table 2:** Genomic variance analysis of asthma symptoms^a^

	V_g_/V_p_	Standard error	*P*-value
All of the SNPs	0.70	0.25	0.001
All of the SNPs, except for the 20 most associated with the outcome.	0.69	0.25	0.001
Only the 20 SNPs most associated to the GWA study	0.12	0.04	0

^a^Corrected by sex and the first three principal components of ancestry

### Enrichment analysis

This analysis is based on prior knowledge of the genes involved in known biological pathways, testing the association between them with the phenotype of interest. All of the metabolic pathways were examined, with 20 presenting empirical *p*-values of less than 0.05 and the haematopoiesis pathway which had an empirical *p*-value of less than 10^−3^: (GO:0030097, p-empirical: 7.9 × 10^−4^). However, these pathways lost statistical significance following multiple test correction (Table 3).

**Table 3:** Metabolic pathways associated with asthma symptoms suggested by enrichment analysis

Metabolic pathway	Total number of genes in the interval	N° of associated genes in the interval	Genes	*p*-empirical	*p*-corrected
GO:0030097: haematopoiesis	52	7	*CD164* (chr6), *KIRREL3* (chr11), *BRCA2* (chr13), *RPA1* (chr17), *BCL11A* (chr2), *PKNOX1* (chr21), *IKZF1* (chr7).	7.90 × 10^−04^	0.76
GO:0070935: 3′-UTR-mediated mRNA stabilization	3	2	*TARDBP* (chr1), *ELAVL1* (chr19).	1.39 × 10^−03^	0.94
GO:001961: flagellum	1	4	*CATSPERB* (chr11), *SPAG16* (chr2), *SPAG17* (chr1), *CATSPER1* (chr14).	1.39 × 10^−03^	0.94
GO:0043922: negative regulation by host of viral transcription	4	2	*POU2F3* (chr11),*TARDBP* (chr1).	1.79 × 10^−03^	0.94
GO:000369: DNA clamp loader activity	5	2	*RFC2* (chr7), *RFC5* (chr12).	4.19 × 10^−03^	0.99
GO:0030212: hyaluronan metabolic process	6	2	*ITIH5* (chr10), *ITIH2* (chr10).	4.19 × 10^−03^	0.99
GO:0050291: sphingosine N-acyltransferase activity	5	2	*LASS4* (chr19), *LASS3* (chr15).	4.59 × 10^−03^	0.99
GO:0005663: DNA replication factor C complex	6	2	*RFC2* (chr7), *RFC5* (chr12).	5.19 × 10^−03^	0.99
GO:004649: S-adenosylhomocysteine metabolic process	6	2	*TPMT* (chr6), *DNMT3A* (chr2).	5.19 × 10^−03^	0.99
GO:0006297: nucleotide-excision repair, DNA gap filling	17	3	*RPA1* (chr17), *RFC5* (chr12), *RFC2* (chr7).	5.79 × 10^−03^	1

## Discussion

We have carried out a GWA study of asthma symptoms in 1246 children in the population of Salvador, Brazil. The 14q11 and 15q22 regions were associated with asthma symptoms.

The 14q11 region has already been reported in different GWA studies associated with dental development [10], obesity [11], narcolepsy [12] and cancer [13]. However, this association in asthma studies had not yet been reported. We analysed the LD between rs1999071 and each of the SNPs on 14q11 region presented in those publications, but none of them were in LD (r^2^ ≥ 0.80) with rs1999071 in our population. If rs1999071 is involved in asthma pathogenesis, then it is unlikely to represent a shared aetiology with the conditions above.

Studies on candidate genes in the 14q11 region found association with SNPs in genes involved in the modulation of inflammatory and immunological responses. The *LTB4 (leukotriene beta 4 receptor)* gene was associated to asthma [14] and *TRA* (T cell alpha receptor) associated with a skin prick test (SPT) in a linkage study in a group of asthmatic families [15]. Furthermore, based on described biological functions, it is reasonable to suppose that genes which are potentially associated with asthma symptoms may be located in this region, with the example of SLC7A7, MMP14 and DAD1. The *SLC7A7* gene is involved in the macrophage differentiation process [16] and its involvement in asthma pathogenesis has been described [17]. *MMP14* is involved with remodelling the extracellular matrix [18] and, specifically, the remodelling of the airway epithelium [19]. *DAD1* is active in the apoptosis regulation process [20] and its failure in this process may lead to increased lymphocytes in asthma patients [21]. The variant which was most associated in this study, rs1999071, is located in the region flanking the *OXA1L* gene that encodes a component of the evolutionarily conserved Oxa1/Alb3/YidC protein family, which is involved in the biogenesis of membrane proteins of mitochondria, chloroplasts and bacteria [22]. Although asthma is not considered a mitochondrial syndrome, there is a considerable overlap between asthma pathophysiology and mitochondrial biology in aspects of apoptosis, oxidative stress and homeostasis of calcium ions [23]. Alterations to oxidative stress may lead to developing asthma by activating pro-inflammatory pathways [24]. Alteration of the Ca^++^ homeostasis in the bronchial smooth muscle cells increases mitochondrial biogenesis, cellular proliferation and, consequently, remodelling of the airways in asthmatic patients [25].

The second most associated region in this study was 15q21, the rs10519031 flanks the *FOXB1* gene which belongs to the family of FOX (forkhead box) transcription factors, with more than 40 members expressed in mammals. Mutations in this group of genes have important effects on human diseases [26]. However, the FOXB1 protein has only been described as being involved in regulating embryonic development [27] until this time.

The 15q21 has already been described in GWA of asthma, with the most associated genes being *RORA, SMAD3* and *SCG3* [28]. RORA is a transcription factor which belongs to the nuclear hormone receptor (NR1) superfamily and links as monomers to specific hormonal response elements in the DNA [29]. It may increase or restrain the transcription of target genes [30] and is differentially expressed during development of the human lung. SMAD3 (SMAD protein family member 3) is a (later) downstream transcription factor of TGFβ and is important for metabolic pathways of regulatory T cells and TH17 [31] cells. It is related to the metabolic pathway of regulatory T cells which forms part of the common [32] process of negative regulation of TH1 and TH2 [33]. *SCG3* (secretogranin 3) encodes a protein member of the neuroendocrine secretory protein family, chromogranin/secretogranin, which are ubiquitous protein regulators of protein secretion [34]. However, there has been little research on its functions.

An important disagreement between our study and previous GWAs findings was the absence of association in the 17q21 region [5] with asthma symptoms. However, the power of our study was limited by the sample size of 280 cases and 966 controls, and we may simply have been underpowered to detect previously known SNPs. Our limited sample size probably accounts also for the high effect sizes of the associated SNPs in our study, ranging from 1.78 to 3.0; while our observed associations were genome-wide significant, they were probably biased upwards by the “winner’s curse” effect [35] . Independent replication is needed to confirm these associations and accurately estimate their effect sizes. We did not achieve compelling replication in Mexican and Latino United States cohorts, but this could have been affected by differences in phenotype definition, sample ancestry, available SNPs and sample size.

For the majority of complex diseases, the associated SNPs from genome-wide association studies (GWAs) only explain a small fraction of heritability. The estimate of the variance explained in liability to asthma symptoms was 70 % in this article, which is a high but also consistent with previous findings in family studies [36] and in cohort studies [37]. These results reinforce the idea that asthma is a complex disease with polygenic inheritance in which individually different genes and their polymorphisms contribute very little to the outcome, but there is a major effect when they are analysed together. Analysis with GCTA explained a substantial proportion of the “missing heritability” and provided evidence that the additive genetic influence of various common SNPs is a powerful determinant of childhood asthma.

It is important to understand that genome-wide studies have analytical limitations, such as not detecting rare variants. Therefore, other complementary approaches are needed such as resequencing, gene expression analysis and replication in other populations. The main limitation of this study is related to power, as the number of cases was relatively small in this prospective cohort, and does not, for example, allow us to differentiate atopic from non-atopic asthma. The sample used was considered adequate for classic epidemiological studies but genome-wide or enrichment studies require a larger sample population than in classical analyses and it is possible that no metabolic pathway associated to asthma symptoms was found as a result.

## Conclusions

Finally, it is concluded that the 14q11 and 15q21 regions may be associated with asthma symptoms in childhood in the population studied. In addition, eQTL analysis suggests that rs1999071 at 14q21, associated with asthma in this study, regulates the expression of *OXA1L* in lung tissue. But these regions explain less than 12 % of variation in liability to this phenotype. A total of 70 % of variation in liability may be explained by common genetic variants, confirming the polygenic nature of asthma.

## Methods

### Study design and characteristics of the population

The data analysed here on asthma and genetic markers were collected in 2 005, as part of the Social Changes, Asthma and Allergy in Latin America (SCAALA) project*.* The SCAALA composes the EPIGEN-Brazil initiative, it is based on three well-defined ongoing population-based cohorts from Brazil’s regions [38]. The design of the original cohort and data collection for asthma are described in detail elsewhere [39]. The sample in this analysis comprises 1 307 children, between 5 and 12 years old, who are resident in the city of Salvador, State of Bahia, Brazil. The city has more than 2.6 million inhabitants and 80 % of the population declare themselves as black or of mixed race [40].

### Data collection

A questionnaire based on the second phase of the ISAAC [41] study was used, with questions on asthma symptoms which had been translated into Portuguese and applied by appropriately trained researchers during home visits. The interviews were carried out with the children’s mother, father or caregiver, provided that the person providing the information knew how to describe the possible presence of signs and symptoms compatible with asthma. Written informed consent was obtained from the legal guardian of each subject. The project was approved by the ethics committees at the Federal University of Bahia (register 003-05/CEP-ISC) and National Council for Ethics in Research (CONEP, resolution number 15 895/2011).

### Definition of asthma symptoms

The children were classified as asthmatic when the parents or caregiver reported wheezing in the 12 months prior to applying the questionnaire associated with any one of the following situations: diagnosis of asthma by a doctor at any time in their lives, wheezing with exercise in the last 12 months, four or more episodes of wheezing in the 12 months or waking up at night due to wheezing episodes in the last 12 months. This definition is more specific than using only wheezing in the last 12 months, more commonly reported by studies using the ISAAC questionnaire. All the other children not fulfilling these criteria were classified as non-asthmatic.

### Genotyping and quality control

The genotyped SNPs were carried out with an Illumina HumanOmni2.5-8v1 Kit BeadChip (Illumina, San Diego, CA) commercial panel with 2 284 818 SNPs. One individual was excluded from the analysis due to inconsistency between the sex registered and the genetic sex, based on X chromosome SNPs. Sixty-one individuals were removed from the sample due to the relationship determined by kinship coefficients for each possible pair of individuals. This method is implemented in the REAP software (Relatedness Estimation in Admixed Populations) [42]. We considered a pair of individuals as related if the estimated kinship coefficient between them was ≥0.1. This cut-off includes second- degree relatives such as a person’s uncle/aunt, nephew/niece, grandparent/grandchild or half- sibling, and any closer pair of relatives.

Quality control was carried out in stages (Additional file 1: Table S1): a genotyping call rate of less than 0.98; deviance in the Hardy-Weinberg equilibrium, with a *p*-value of less than 10^−4^ and Minor Allele Frequency *(MAF*) of less than 1 % [43].

### Replication studies

#### Genes-environments & Admixture in Latino Americans study (GALA II)

The Genes-environments & Admixture in Latino Americans (GALA II) study is an ongoing multicenter case–control study of asthma in Latino children and adolescents, organized from the coordinating center based at the University of California, San Francisco. It is comprised of 3 774 participants (1 893 asthma cases and 1 881 controls). GALA II recruited Latinos from urban regions in the mainland United States (Chicago, IL; Bronx, NY; Houston, TX; San Francisco Bay Area, CA) and Puerto Rico, using a combination of community and clinic-based recruitment. Subjects were eligible if they were 8–21 years of age, self-identified all four grandparents as Latino, and had <10 pack-years of smoking history. Asthma was defined based on physician diagnosis and report of symptoms and medication use within the last two years prior to the recruitment [44].

### Mexico City Childhood Asthma Study (MCCAS)

This is a case-parent trio design where the population from Mexico City Childhood Asthma Study (MCCAS) has been previously described [45]. Genome wide association data were available on 498 children between the ages of 5–17 with asthma and their parents. Subjects were recruited between June 1998 and November 2003 from a paediatric allergy specialty clinic at a public hospital in central Mexico City. The childhood asthma was diagnosed by allergists at the referral clinic, according to the guidelines of the British Thoracic Society and Scottish Intercollegiate Guidelines Network.

### Statistical analysis

#### Genome-Wide Association

Logistic regression was used to examine the association with asthma symptoms with an additive genetic model. Conventionally, an association is considered suggestive when the *p*-value is between 10^−6^ and 5 × 10^−8^ and genome-wide significantly when the *p*-value is less than 5 × 10^−8^. Principal Component Analysis was carried out and its first three components were used as covariates to control confounding by population structure. In addition the genomic inflation factor (λ) was calculated, in order to visualise and avoid inflated test statistics in the results [46]. Replication of the original finding was defined as a *p*-value of less than 0.05 with an effect in the same direction as in the GWAS. Fixed effects meta-analysis of the SCAALA and GALA II studies was performed by the GWAMA software [47]. Only *p*-values were available from MCCAS so Fisher’s combined *p*-values were calculated for the meta-analysis of SCAALA, GALA II and MCCAS.

#### SNP imputation

The genotypes were imputed, only in regions of interest, using the IMPUTE2 package [48] on the public panel from 1000 Genomes Project Phase I data “version 3” (ALL.integrated_phase1_SHAPEIT_16-06-14.nomono.integrated_phase1_v3.20101123.snps_indels_svs.genotypes.nomono.haplotypes.gz) [49], which contained 1092 individuals of various ethnicities. Quality control was carried out once more following imputation and the SNPs which presented a MAF lower than 1 %, a deviance in the Hardy-Weinberg equilibrium (*p* <10^−4^) or had a genotyping call rate of under 95 % were excluded.

#### Heritability estimate

The proportion of variance in liability for all of the SNPs was estimated as (V_g_/V_p_) in which V_g_ is the variance component attributable to genetic variation in the genotyped SNPs and V_p_ is the total phenotypic variance observed. The GCTA software package was used, which uses genetic variant data to estimate additive genetic relationships (correlations) between distantly related individuals. The method treats the total effect of all of the SNPs as a random effect in a Mixed Linear Model (MLM) [50]. The variance of this random effect is an estimate of V_g_. This analysis was adjusted for sex and first three principal components.

#### Enrichment analysis based on a defined set of genes

An aggregation analysis was carried out, based on linkage disequilibrium in order to identify a list of genic regions associated to the outcome (parameters for PLINK = clump-p1 = 0.005; clump-p2 = 0.05; clump-r2 = 0.5; clump-kb = 250). Regions 20 kb up/downstream from the initial and final transcription sites for 17 529 genes in the autosomal chromosomes were then defined, according to the GRCh37/hg19 public database of catalogued genes. We performed enrichment analysis using the INRICH [51] program, comprising two stages. The number of times that the genomic intervals, identified a priori, including a set of predetermined genes is counted in the first stage. A second stage was carried out to correct the false-positive rate, using a permutation procedure based on 1000 repetitions in order to obtain the empirical *p*-value, representing the proportion of times that this genomic interval includes a specific gene.

## Additional file

Additional file 1: Figure S1. Analysis of the principal components in the SCAALA population with all of the SNPs in order to deduce population structure. **Table S2.** The 100 SNPs that are most associated with childhood asthma symptoms. **Table S3.** Combined analysis. **Figure S2.** Phenotypic variance explained for each chromosome. **Table S1.** Quality Control steps for SNPs. (DOCX 125 kb)

## Abbreviations

GWAS: Genome-Wide Association Studies
SNPs: Single Nucleotide Polymorphisms
MAF: Minor allele frequency
OR: Odds Ratio
95 %CI: 95 % Confidence Interval
SPT: Skin Prick Test
PCA: Principal Component Analysis
GTEx: Genotype-Tissue Expression
GO: Gene Ontology
GCTA: Genome-wide Complex Trait Analysis
SCAALA: Social Changes, Asthma and Allergy in Latin America
ISAAC: International Study of Asthma and Allergies in Childhood
GALA II: Genes-environments & Admixture in Latino Americans
MCCAS: Mexico City Childhood Asthma Study
