# Supplementary material for: A genome-wide association study of asthma symptoms in Latin American children
Source: BMC Genet. 2015 Dec 3;16:141. doi: 10.1186/s12863-015-0296-7 (PMC4669662; doi:10.1186/s12863-015-0296-7)
Supplement: Additional file 1: Figure S1. — Analysis of the principal components in the SCAALA population with all of the SNPs in order to deduce population structure. Table S2. The 100 SNPs that are most associated with childhood asthma symptoms. Table S3. Combined analysis. Figure S2. Phenotypic variance explained for each chromosome. Table S1. Quality Control steps for SNPs. (DOCX 125 kb) [file 12863_2015_296_MOESM1_ESM.docx]

**Supplementary material**

**Genotyping and control quality for replication analysis:**

**Mexico City Childhood Asthma Study**: A total of 498 complete case-parent trios with previously confirmed parentage and DNA were genotyped for 561,466 SNPs using the Illumina HumanHap 550K beadChip, version 3. DNA was extracted using Gentra Puregene kits (Gentra Systems, Minneapolis, Minnesota). The subjects studied were genotyped with an average call rate of 99.7%, missingness, 0.1%, MAF, 0.001%, HWE p-value (in parents only),10^-10^, Mendelian errors in more than two families and heterozygous genotype calls for chromosome X SNPs in more than one male[[1](#_ENREF_1), [2](#_ENREF_2)].

**Genes-environments & Admixture in Latino Americans (GALA II) study:** GALA II subjects were genotyped using the Axiom LAT1 array (World Array 4, Affymetrix, Santa Clara, Calif) which includes 817,810 single nucleotide polymorphisms (SNPs) and was specifically designed to capture genetic variation in Latino populations. SNPs that did not pass quality control procedures were removed from the analyses using the following criteria: failing Axiom quality controls, <95% call rate or deviation from Hardy-Weinberg equilibrium (*p*<10^-6^) within each defined population (Puerto Rican, Mexican, and other Latino). Therefore, genotypes for 723,888 high-quality autosomal SNPs were available for the analyses. SNPs not directly genotyped were imputed using all populations available from 1000 Genomes Project Phase I v3 (ref: The 1000 Genomes Project Consortium. Nature 2010; 467:1061-73) as reference by first phasing the genotypes using the software SHAPE- IT[[3](#_ENREF_3)] and then imputing with IMPUTE2. Association testing was performed using PLINK (ref. Purcell S. PLINK: a toolset for whole-genome association and population-based linkage analysis. American Journal of Human Genetics, 81) only for those variants that had a high quality imputation score (info score ≥0.4)[[4](#_ENREF_4), [5](#_ENREF_5)].

**Table S1:** Quality Control steps for SNPs.

| **Control quality steps** | **cutoff** | **nº of SNPs** |
| --- | --- | --- |
| MAF | < 1% | 354,422 |
| HW | p-value < 10^-4^ | 3597 |
| Low genotyping for SNPs | > 10% | 365 |
| Chromosomes X and Y | excluded all | 47.336 |

**Table S2**: The 100 SNPs that are most associated with childhood asthma symptoms.

| **Rank** | **Chromosome** | **SNP** | **Position (bp)** | **Risk allele** | **Gene** | **Odds ratio** | **CI(95%)** | ***p*** |
| --- | --- | --- | --- | --- | --- | --- | --- | --- |
| 1 | 14 | rs1999071 | 23129207 | C | INTERGENIC | 1.782 | (1.453 – 2.185) | 2.834E-08 |
| 2 | 15 | rs10519031 | 60183005 | C | INTERGENIC | 3.008 | (2.017 – 4.487) | 6.676E-08 |
| 3 | 15 | rs8029377 | 60191985 | C | INTERGENIC | 2.495 | (1.763 – 3.53) | 2.454E-07 |
| 4 | 3 | rs77165709 | 159452876 | T | IQCJ-SCHIP1 | 2.275 | (1.661 – 3.117) | 3.045E-07 |
| 5 | 10 | rs10159952 | 77562470 | A | C10orf11 | 2.041 | (1.533 – 2.717) | 1.016E-06 |
| 6 | 9 | rs1329568 | 37037976 | T | LOC100130458 | 1.933 | (1.473 – 2.537) | 2.02E-06 |
| 7 | 7 | rs1425883 | 49754984 | T | INTERGENIC | 0.6043 | (0.4908 – 0.7439) | 2.037E-06 |
| 8 | 7 | rs1543902 | 49754752 | G | INTERGENIC | 0.6044 | (0.4907 – 0.7444) | 2.164E-06 |
| 9 | 18 | rs76227669 | 10787366 | T | PIEZO2 | 4.063 | (2.241 – 7.366) | 3.881E-06 |
| 10 | 9 | rs4878674 | 37036329 | T | INTERGENICX5 | 1.9 | (1.446 – 2.497) | 4.027E-06 |
| 11 | 10 | rs1244495 | 7881339 | G | TAF3 | 0.6137 | (0.4985 – 0.7555) | 4.169E-06 |
| 12 | 1 | rs269330 | 68548590 | T | GNG12-AS1 | 1.749 | (1.377 – 2.221) | 4.669E-06 |
| 13 | 7 | rs10268364 | 28708465 | C | CREB5 | 1.593 | (1.305 – 1.945) | 4.671E-06 |
| 14 | 7 | rs41335 | 28704468 | C | CREB6 | 0.6373 | (0.5254 – 0.773) | 4.793E-06 |
| 15 | 4 | rs72998173 | 173715118 | G | GALNTL6 | 1.758 | (1.38 – 2.239) | 4.848E-06 |
| 16 | 3 | rs4373023 | 34077223 | T | INTERGENIC | 1.57 | (1.294 – 1.906) | 4.977E-06 |
| 17 | 9 | rs1329567 | 37038326 | A | LOC100130458 | 1.969 | (1.47 – 2.636) | 5.46E-06 |
| 18 | 9 | rs2381598 | 37041246 | C | INTERGENIC | 1.968 | (1.47 – 2.636) | 5.474E-06 |
| 19 | 15 | rs12901887 | 56941976 | G | ZNF280D | 0.5567 | (0.4318 – 0.7177) | 6.224E-06 |
| 20 | 1 | rs79530846 | 171950308 | T | vDNM3 | 3.078 | (1.882 – 5.035) | 7.542E-06 |
| 21 | 4 | rs6535350 | 83439160 | G | TMEM150C | 1.593 | (1.299 – 1.954) | 7.816E-06 |
| 22 | 15 | rs1968687 | 58822697 | G | LIPC | 1.542 | (1.275 – 1.864) | 7.826E-06 |
| 23 | 2 | rs113750103 | 202398743 | T | ALS2CR11 | 2.391 | (1.629 – 3.509) | 8.431E-06 |
| 24 | 1 | rs78521816 | 171414740 | A | INTERGENIC | 2.299 | (1.594 – 3.317) | 8.483E-06 |
| 25 | 7 | rs114576775 | 54243876 | A | INTERGENIC | 4.023 | (2.179 – 7.428) | 8.642E-06 |
| 26 | 3 | rs80316500 | 159982507 | C | IFT80 | 1.594 | (1.298 – 1.958) | 8.742E-06 |
| 27 | 20 | rs1555852 | 19536127 | T | SLC24A3 | 1.72 | (1.354 - 2.186) | 8.959E-06 |
| 28 | 7 | rs78980017 | 54227948 | G | INTERGENIC | 4.008 | (2.171 - 7.4) | 9.092E-06 |
| 29 | 5 | rs10069226 | 31271333 | A | CDH6 | 2.344 | (1.606 – 3.421) | 1.013E-05 |
| 30 | 9 | rs4880052 | 37036285 | A | INTERGENIC | 1.893 | (1.425 – 2.515) | 1.078E-05 |
| 31 | 3 | rs7622659 | 45357777 | A | INTERGENIC | 2.324 | (1.596 – 3.383) | 1.083E-05 |
| 32 | 15 | rs35743173 | 101324457 | A | LOC440313 | 2.498 | (1.661 – 3.756) | 1.09E-05 |
| 33 | 11 | rs7928089 | 68574429 | A | CPT1A | 1.55 | (1.275 – 1.886) | 1.118E-05 |
| 34 | 15 | rs4775232 | 60310375 | C | INTERGENIC | 1.525 | (1.263 – 1.842) | 1.143E-05 |
| 35 | 3 | rs9827639 | 45342749 | T | INTERGENIC | 2.372 | (1.612 – 3.49) | 1.159E-05 |
| 36 | 20 | rs942992 | 19564963 | T | SLC24A3 | 1.787 | (1.378 – 2.316) | 1.161E-05 |
| 37 | 7 | rs58285948 | 7305712 | C | INTERGENIC | 2.222 | (1.555 – 3.176) | 1.172E-05 |
| 38 | 3 | rs9815657 | 45345904 | T | INTERGENIC | 2.37 | (1.611 – 3.486) | 1.187E-05 |
| 39 | 1 | rs78063878 | 172018390 | G | DNM3 | 3.126 | (1.875 – 5.211) | 1.233E-05 |
| 40 | 3 | rs6767038 | 159453369 | T | SCHIP1 | 1.561 | (1.278 – 1.906) | 1.245E-05 |
| 41 | 7 | rs77188806 | 64926662 | T | INTERGENIC | 4.664 | (2.337 – 9.307) | 1.252E-05 |
| 42 | 9 | rs1948308 | 87616257 | T | NTRK2 | 0.6569 | (0.544 – 0.7932) | 1.259E-05 |
| 43 | 1 | rs17097444 | 69918002 | C | INTERGENIC | 3.487 | (1.988 – 6.116) | 1.316E-05 |
| 44 | 14 | rs1681581 | 23118663 | G | INTERGENIC | 1.694 | (1.335 – 2.149) | 1.435E-05 |
| 45 | 1 | rs116375634 | 154273469 | T | INTERGENIC | 3.085 | (1.853 – 5.136) | 1.482E-05 |
| 46 | 6 | rs113623387 | 43162490 | G | CUL9 | 1.535 | (1.264 – 1.864) | 1.51E-05 |
| 47 | 12 | rs78201583 | 20700554 | C | PDE3A | 2.644 | (1.701 – 4.109) | 1.546E-05 |
| 48 | 9 | rs2774241 | 93665453 | A | INTERGENIC | 1.758 | (1.361 – 2.271) | 1.56E-05 |
| 49 | 4 | rs75957247 | 114692320 | T | INTERGENIC | 1.664 | (1.32 – 2.097) | 1.62E-05 |
| 50 | 11 | rs58478357 | 119628335 | C | LOC101929156 | 1.664 | (1.32 – 2.099) | 1.67E-05 |
| 51 | 3 | rs16831149 | 159976271 | T | IFT80 | 1.564 | (1.275 – 1.919) | 1.801E-05 |
| 52 | 15 | rs8042498 | 29605931 | A | FAM189A1 | 2654 | (1.698 – 4.148) | 1.824E-05 |
| 53 | 4 | rs13124037 | 173085238 | T | GALNTL6 | 0.6454 | (0.5283 – 0.7886) | 1.831E-05 |
| 54 | 8 | rs59811079 | 1625732 | G | DLGAP2 | 0.6484 | (0.5317 – 0.7907) | 1.863E-05 |
| 55 | 9 | rs7860117 | 87770429 | G | INTERGENIC | 1.801 | (1.376 – 2.359) | 1.87E-05 |
| 56 | 7 | rs77053500 | 7903345 | A | LOC729852 | 4.725 | (2.319 – 9.629) | 1.907E-05 |
| 57 | 1 | rs115763417 | 30939086 | A | INTERGENIC | 2.705 | (1.714 – 4.271) | 1.928E-05 |
| 58 | 2 | rs77540829 | 202400872 | A | ALS2CR11 | 2.582 | (1.67 – 3.991) | 1.971E-05 |
| 59 | 4 | rs78129004 | 81117017 | A | PRDM8 | 4.149 | (2.159 – 7.975) | 1.975E-05 |
| 60 | 6 | rs2273709 | 43184132 | C | CUL9 | 1.525 | (1.256 – 1.851) | 2E-05 |
| 61 | 17 | rs115798120 | 43405024 | T | INTERGENIC | 1.574 | (1.278 – 1.94) | 2.049E-05 |
| 62 | 2 | rs75681688 | 202383927 | A | ALS2CR11 | 2.623 | (1.682 – 4.091) | 2.111E-05 |
| 63 | 7 | rs982947 | 28708856 | C | CREB5 | 0.6451 | (0.5271 – 0.7896) | 2.122E-05 |
| 64 | 5 | rs17341817 | 103579857 | T | INTERGENIC | 2.543 | (1.654 – 3.911) | 2.126E-05 |
| 65 | 5 | rs11950703 | 103576747 | T | INTERGENIC | 2.543 | (1.654 – 3.911) | 2.126E-05 |
| 66 | 5 | rs2162716 | 94762306 | C | FAM81B | 1.559 | (1.27 – 1.913) | 2.196E-05 |
| 67 | 4 | rs12506762 | 173712840 | T | GALNTL6 | 1.513 | (1.25 – 1.833) | 2.207E-05 |
| 68 | 1 | rs3004245 | 9111626 | A | SLC2A5 | 0.6425 | (0.5237 – 0.7882) | 2.215E-05 |
| 69 | 20 | rs6046176 | 19532518 | C | SLC24A3 | 1.665 | (1.315 – 2.11) | 2.378E-05 |
| 70 | 4 | rs7655284 | 105869702 | G | INTERGENIC | 1.517 | (1.25 – 1.841) | 2.438E-05 |
| 71 | 10 | rs1244480 | 7870324 | T | TAF3 | 1.515 | (1.249 – 1.837) | 2.439E-05 |
| 72 | 9 | rs11140831 | 87647454 | G | INTERGENIC | 0.6083 | (0.4828 – 0.7663) | 2.463E-05 |
| 73 | 3 | rs74887681 | 52890196 | T | TMEM110-MUSTN1 | 5.34 | (2.448 – 11.65) | 2.557E-05 |
| 74 | 2 | rs114446331 | 43225774 | C | INTERGENIC | 3.541 | (1.965 – 6.379) | 2.567E-05 |
| 75 | 8 | rs115506651 | 56047932 | G | XKR4 | 3.407 | (1.924 – 6.032) | 2.616E-05 |
| 76 | 15 | rs115302036 | 60183951 | T | INTERGENIC | 3.186 | (1.856 – 5.468) | 2.626E-05 |
| 77 | 11 | rs7127256 | 90525490 | A | INTERGENIC | 1.507 | (1.245 – 1.825) | 2.652E-05 |
| 78 | 7 | rs7780465 | 7076459 | T | INTERGENIC | 1.538 | (1.258 – 1.881) | 2.652E-05 |
| 79 | 13 | rs56087514 | 27778960 | C | INTERGENIC | 0.6346 | (0.5133 – 0.7846) | 2.654E-05 |
| 80 | 1 | rs80295800 | 235771726 | G | GNG4 | 1.925 | (1.418 – 2.614) | 2.718E-05 |
| 81 | 9 | rs424539 | 14442595 | C | INTERGENIC | 0.6509 | (0.5323 – 0.7958) | 2.833E-05 |
| 82 | 3 | rs116448590 | 142841697 | G | CHST2 | 5.27 | (2.417 – 11.49) | 2.929E-05 |
| 83 | 2 | rs16986498 | 19665812 | G | LOC101928196 | 0.4067 | (0.2665 – 0.6204) | 2.983E-05 |
| 84 | 9 | rs7873362 | 74918896 | T | LINC01504 | 1.765 | (1.352 – 2.306) | 3.031E-05 |
| 85 | 7 | rs6956273 | 63435537 | T | INTERGENIC | 1.6 | (1.283 – 1.995) | 3.046E-05 |
| 86 | 14 | rs1465118 | 68032874 | A | PLEKHH1 | 1.508 | (1.243 – 1.829) | 3.074E-05 |
| 87 | 15 | rs10518870 | 57017888 | G | ZNF280D | 0.5888 | (0.459 – 0.7554) | 3.085E-05 |
| 88 | 7 | rs115972708 | 65372483 | A | VKORC1L1 | 4.399 | (2.187 – 8.85) | 3.261E-05 |
| 89 | 5 | rs73767458 | 82346803 | A | INTERGENIC | 2.574 | (1.648 – 4.022) | 3.287E-05 |
| 90 | 6 | rs9472022 | 43152573 | G | CUL9 | 1.506 | (1.241 – 1.828) | 3.357E-05 |
| 91 | 10 | rs79846008 | 128542978 | A | *LOC728065* | 2.679 | (1.681 – 4.268) | 3.368E-05 |
| 92 | 4 | rs74426337 | 170929983 | T | MFAP3L | 3.031 | (1.793 – 5.123) | 3.477E-05 |
| 93 | 9 | rs4419891 | 87609384 | T | NTRK2 | 1.531 | (1.251 – 1.872) | 3.477E-05 |
| 94 | 4 | rs77924825 | 170923535 | C | MFAP3L | 3.027 | (1.791 – 5.118) | 3.538E-05 |
| 95 | 8 | rs2952238 | 10095328 | A | MSRA | 1.735 | (1.336 – 2.253) | 3.587E-05 |
| 96 | 3 | rs76336861 | 45368350 | G | INTERGENIC | 2.055 | (1.46 – 2.892) | 3.61E-05 |
| 97 | 20 | rs529058 | 926945 | A | INTERGENIC | 2.098 | (1.475 – 2.982) | 3.678E-05 |
| 98 | 1 | rs74081353 | 68544206 | G | LOC100289178 | 2.02 | (1.446 – 2.821) | 3.696E-05 |
| 99 | 3 | rs9867680 | 45361222 | T | INTERGENIC | 2.046 | (1.455 – 2.878) | 3.917E-05 |
| 100 | 6 | rs6914964 | 43146387 | G | SRF | 1.615 | (1.285 – 2.029) | 3.927E-05 |

**Table** **S3**: Combined analysis.

|  |  | **SCAALA** | | | **GALA II** | | | **Combined¹** | | | **MCCA** |
| --- | --- | --- | --- | --- | --- | --- | --- | --- | --- | --- | --- |
| **Chromosome** | **SNP** | **Risk allele** | **OR** | **p-value** | **Risk allele** | **OR** | **p-value** | **OR combined** | **p-value**  **combined** | **Effect** | **p-value** |
| 1 | rs269330 | T | 1.75 | 4.67E-06 | T | 1.130 | 0.289 | 1.40 | 0.12 | ++ | 0.24 |
| 1 | rs78521816 | T | 2.30 | 8.48E-06 | A | 1.000 | 0.999 | 1.53 | 0.31 | +- | - |
| 1 | rs79530846 | A | 3.08 | 7.54E-06 | T | 1.084 | 0.783 | 1.85 | 0.24 | ++ | - |
| 2 | rs113750103 | T | 2.39 | 8.43E-06 | T | 0.756 | 0.158 | 1.35 | 0.61 | +- | - |
| 3 | rs4373023 | T | 1.57 | 4.98E-06 | C | 1.040 | 0.520 | 1.22 | 0.42 | +- | 0.40 |
| 3 | rs77165709 | T | 2.28 | 3.05E-07 | T | 0.907 | 0.507 | 1.43 | 0.43 | +- | - |
| 4 | rs6535350 | G | 1.59 | 7.82E-06 | G | 0.900 | 0.168 | 1.19 | 0.54 | +- | 0.47 |
| 4 | rs72998173 | G | 1.76 | 4.85E-06 | G | 1.119 | 0.130 | 1.39 | 0.15 | ++ | - |
| 7 | rs10268364 | C | 1.59 | 4.67E-06 | C | 1.059 | 0.345 | 1.29 | 0.21 | ++ | - |
| 7 | rs114576775 | A | 4.02 | 8.64E-06 | A | 1.161 | 0.705 | 2.21 | 0.20 | ++ | - |
| 7 | rs1425883 | T | 0.60 | 2.04E-06 | C | 1.041 | 0.448 | 0.77 | 0.26 | -- | - |
| 7 | rs1543902 | G | 0.60 | 2.16E-06 | T | 1.039 | 0.468 | 0.77 | 0.26 | -- | 0.36 |
| 7 | rs41335 | C | 0.64 | 4.79E-06 | G | 1.032 | 0.560 | 0.79 | 0.27 | -- | - |
| 9 | rs1329567 | A | 1.97 | 5.46E-06 | G | 0.999 | 0.995 | 1.40 | 0.32 | ++ | 0.43 |
| 9 | rs1329568 | T | 1.93 | 2.02E-06 | G | 0.976 | 0.838 | 1.40 | 0.29 | ++ | - |
| 9 | rs2381598 | C | 1.97 | 5.47E-06 | T | 1.002 | 0.986 | 1.40 | 0.33 | +- | 0.43 |
| 9 | rs4878674 | T | 1.90 | 4.03E-06 | G | 0.972 | 0.803 | 1.39 | 0.28 | ++ | - |
| 10 | **rs10159952** | A | 2.04 | 1.02E-06 | A | 1.370 | **0.014** | 1.66 | **0.01** | ++ | 0.49 |
| 10 | rs1244495 | G | 0.61 | 4.17E-06 | - | - | - | - | - | - | 0.25 |
| 14 | rs10131634 | T | 1.66 | 1.39E-06 | T | 1.036 | 0.524 | 1.32 | 0.25 | ++ | - |
| 14 | rs10131924 | A | 1.66 | 1.51E-06 | A | 1.036 | 0.525 | 1.31 | 0.25 | ++ | - |
| 14 | rs10143684 | T | 1.64 | 2.50E-06 | T | 1.042 | 0.453 | 1.31 | 0.23 | ++ | - |
| 14 | rs10144348 | T | 1.66 | 1.39E-06 | T | 1.032 | 0.560 | 1.31 | 0.25 | ++ | - |
| 14 | rs10151422 | C | 1.67 | 1.05E-06 | C | 1.036 | 0.516 | 1.32 | 0.24 | ++ | - |
| 14 | rs1242937 | T | 1.70 | 1.53E-05 | T | 1.069 | 0.332 | 1.35 | 0.19 | ++ | - |
| 14 | rs1242938 | T | 1.61 | 6.58E-05 | T | 1.067 | 0.334 | 1.32 | 0.18 | ++ | - |
| 14 | rs1242940 | T | 1.61 | 6.58E-05 | T | 1.065 | 0.353 | 1.32 | 0.18 | ++ | 0.24 |
| 14 | rs1242941 | C | 1.65 | 3.89E-06 | C | 1.049 | 0.413 | 1.32 | 0.22 | ++ | - |
| 14 | rs1242942 | T | 1.62 | 5.70E-06 | T | 1.038 | 0.522 | 1.30 | 0.24 | ++ | - |
| 14 | rs1681581 | G | 1.67 | 2.48E-05 | G | 1.066 | 0.357 | 1.34 | 0.19 | ++ | - |
| 14 | rs1681582 | C | 1.61 | 7.89E-05 | C | 1.065 | 0.353 | 1.32 | 0.18 | ++ | - |
| 14 | rs1958322 | G | 1.71 | 6.02E-07 | G | 1.104 | 0.101 | 1.38 | 0.14 | ++ | - |
| 14 | rs1999070 | G | 1.70 | 1.53E-07 | G | 1.066 | 0.266 | 1.35 | 0.20 | ++ | - |
| 14 | rs1999071 | C | 1.78 | 2.83E-08 | C | 1.059 | 0.318 | 1.36 | 0.23 | ++ | 0.28 |
| 14 | rs28523687 | C | 1.69 | 7.24E-07 | C | 1.072 | 0.228 | 1.35 | 0.19 | ++ | - |
| 14 | rs34222671 | C | 1.62 | 3.87E-06 | C | 1.036 | 0.516 | 1.30 | 0.24 | ++ | - |
| 14 | rs34226211 | T | 1.62 | 4.18E-06 | T | 1.036 | 0.518 | 1.30 | 0.24 | ++ | - |
| 14 | rs34437928 | A | 1.65 | 1.75E-06 | A | 1.036 | 0.520 | 1.31 | 0.24 | ++ | - |
| 14 | rs35622014 | T | 1.64 | 2.82E-06 | T | 1.035 | 0.534 | 1.31 | 0.24 | ++ | - |
| 14 | rs35622551 | G | 1.76 | 2.45E-07 | G | 1.098 | 0.123 | 1.40 | 0.16 | ++ | - |
| 14 | rs56861039 | C | 1.66 | 1.47E-06 | C | 1.035 | 0.533 | 1.31 | 0.25 | ++ | - |
| 14 | rs8008677 | G | 1.65 | 1.42E-06 | G | 1.036 | 0.517 | 1.31 | 0.24 | ++ | - |
| 15 | rs10519031 | C | 3.01 | 6.68E-08 | C | 0.834 | 0.527 | 1.61 | 0.46 | +- | 0.34 |
| 15 | rs112012489 | C | 2.72 | 2.85E-06 | C | 0.838 | 0.539 | 1.57 | 0.45 | +- | - |
| 15 | rs115302036 | T | 3.15 | 3.09E-05 | T | 1.069 | 0.849 | 1.94 | 0.22 | ++ | - |
| 15 | rs116765599 | T | 2.17 | 2.93E-05 | T | 1.267 | 0.287 | 1.74 | 0.04 | ++ | - |
| 15 | rs12901887 | G | 0.56 | 6.22E-06 | G | 0.980 | 0.739 | 0.75 | 0.30 | -- | - |
| 15 | rs139751352 | A | 3.87 | 8.94E-05 | A | 1.646 | 0.225 | 2.79 | 0.01 | ++ | - |
| 15 | rs149023356 | C | 6.35 | 3.78E-05 | C | 1.392 | 0.475 | 3.19 | 0.12 | ++ | - |
| 15 | rs16941890 | C | 2.78 | 1.65E-06 | C | 0.839 | 0.541 | 1.58 | 0.44 | +- | - |
| 15 | rs16942157 | C | 1.75 | 5.25E-05 | C | 0.923 | 0.568 | 1.29 | 0.42 | +- | 0.29 |
| 15 | rs16942159 | C | 1.74 | 7.33E-05 | C | 0.923 | 0.568 | 1.29 | 0.43 | +- | 0.30 |
| 15 | rs180995748 | C | 4.40 | 5.59E-05 | C | 1.420 | 0.453 | 2.75 | 0.07 | ++ | - |
| 15 | rs1968687 | G | 1.54 | 7.83E-06 | T | 1.075 | 0.232 | 1.19 | 0.49 | +- | - |
| 15 | rs201996260 |  | 5.65 | 6.37E-05 | - | - | - | - | - | - | - |
| 15 | rs4775232 | T | 1.52 | 1.23E-05 | T | 1.039 | 0.590 | 1.22 | 0.39 | +- | 0.30 |
| 15 | rs56036906 | A | 1.79 | 4.05E-05 | A | 1.147 | 0.363 | 1.47 | 0.08 | ++ | - |
| 15 | rs56856389 | T | 2.25 | 1.31E-05 | T | 0.924 | 0.744 | 1.49 | 0.37 | +- | - |
| 15 | rs73419027 | G | 2.62 | 1.44E-05 | G | 0.993 | 0.978 | 1.67 | 0.29 | +- | - |
| 15 | rs73419030 | A | 2.66 | 8.79E-06 | A | 0.988 | 0.964 | 1.68 | 0.30 | +- | - |
| 15 | rs76270731 | G | 2.33 | 6.85E-05 | G | 1.065 | 0.738 | 1.61 | 0.22 | ++ | - |
| 15 | rs78573950 | A | 2.62 | 5.69E-06 | A | 0.840 | 0.544 | 1.54 | 0.45 | +- | - |
| 15 | rs8029377 | C | 2.50 | 2.45E-07 | C | 0.927 | 0.756 | 1.54 | 0.38 | +- | - |
| 15 | rs993192 | C | 2.43 | 3.17E-05 | C | 1.074 | 0.703 | 1.65 | 0.22 | ++ | - |
| 18 | rs76227669 | T | 4.06 | 3.88E-06 | T | 0.815 | 0.520 | 1.82 | 0.45 | +- | - |

**In bold:** SNP replicated in GALA II study.

**Figure S1**: Analysis of the principal components in the SCAALA population with all of the SNPs in order to deduce population structure.


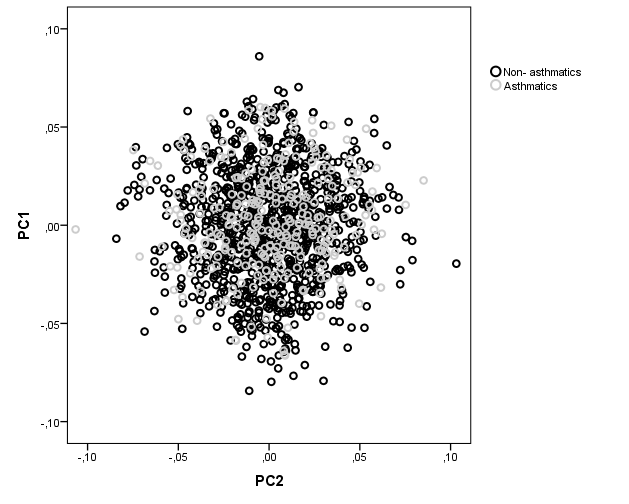


**Figure S2**: Phenotypic variance explained for each chromosome.

# REFERENCE:

# 1. Torgerson DG, Ampleford EJ, Chiu GY, Gauderman WJ, Gignoux CR, Graves PE et al. Meta-analysis of genome-wide association studies of asthma in ethnically diverse North American populations. Nature genetics. 2011;43(9):887-92. doi:10.1038/ng.888

# 2. Hancock DB, Romieu I, Shi M, Sienra-Monge JJ, Wu H, Chiu GY et al. Genome-wide association study implicates chromosome 9q21.31 as a susceptibility locus for asthma in mexican children. PLoS genetics. 2009;5(8):e1000623. doi:10.1371/journal.pgen.1000623.

# 3. Delaneau O, Marchini J, Zagury JF. A linear complexity phasing method for thousands of genomes. Nature methods. 2012;9(2):179-81. doi:10.1038/nmeth.1785.

# 4. Nishimura KK, Galanter JM, Roth LA, Oh SS, Thakur N, Nguyen EA et al. Early-life air pollution and asthma risk in minority children. The GALA II and SAGE II studies. American journal of respiratory and critical care medicine. 2013;188(3):309-18. doi:10.1164/rccm.201302-0264OC.

# 5. Pino-Yanes M, Thakur N, Gignoux CR, Galanter JM, Roth LA, Eng C et al. Genetic ancestry influences asthma susceptibility and lung function among Latinos. The Journal of allergy and clinical immunology. 2015;135(1):228-35. doi:10.1016/j.jaci.2014.07.053.
